# Prebiotics as modulators of colonic calcium and magnesium uptake

**DOI:** 10.1111/apha.14262

**Published:** 2025-01-13

**Authors:** Friederike Stumpff, David Manneck

**Affiliations:** ^1^ Institute for Molecular Medicine Health and Medical University Potsdam Potsdam Germany

**Keywords:** calcium, fermentation, magnesium, prebiotics, SCFA, TRPV3

## Abstract

Ca^2+^ and Mg^2+^ are essential nutrients, and deficiency can cause serious health problems. Thus, lack of Ca^2+^ and Mg^2+^ can lead to osteoporosis, with incidence rising both in absolute and age‐specific terms, while Mg^2+^ deficiency is associated with type II diabetes. Prevention via vitamin D or estrogen is controversial, and the bioavailability of Ca^2+^ and Mg^2+^ from supplements is significantly lower than that from milk products. Problems are likely to increase as populations age and the number of people on vegan diets surges. Developing new therapeutic strategies requires a better understanding of the molecular mechanisms involved in absorption by intestinal epithelia. The vitamin‐D dependent, active pathway for the uptake of Ca^2+^ from the upper small intestine involving TRPV6 is highly efficient but only accounts for about 20% of total uptake. Instead, most Ca^2+^ uptake is thought to occur via passive paracellular diffusion across the ileum, although sufficiently high luminal concentrations are difficult to achieve.. Interestingly, colon and caecum also have a considerable capacity for the active absorption of Ca^2+^ and Mg^2+^, the molecular mechanisms of which are unclear. Intriguingly, stimulating fermentation by prebiotics enhances colonic absorption, which can rise from ~10% to ~30% of the total. Notably, fermentation releases protons, which inhibits channels highly selective for Ca^2+^ and Mg^2+^ (TRPV6 and TRPM6/TRPM7). Conversely, the non‐selective cation channel TRPV3 is stimulated by both intracellular acidification and by numerous herbal compounds. Spicy, fiber‐rich food, as traditionally consumed in many cultures, might enhance the uptake of Ca^2+^ and Mg^2+^ via this pathway.

## 1 INTRODUCTION

Calcium and magnesium are essential nutrients, required for a multitude of physiological processes. To name but one example, Ca^2+^ and Mg^2+^ are required for bone formation, and an adequate supply is considered to be essential in preventing osteoporosis as a major and growing public health concern.^1^, ^2^ Thus, age‐specific rates of hip fracture in Beijing, China, have increased dramatically and are rapidly approaching the two‐fold higher values found in the USA or Hong Kong.^3^ A common preventative strategy is to enrich the diet with vitamin D. However, many studies suggest that the effects are marginal, lacking, or even detrimental unless specific risk factors for a deficiency are present.^4^, ^5^ Supplemental estrogen is effective but associated with a number of health risks that include higher rates of cancer. Effects of drugs that inhibit bone resorption (such as denosumab) or increase bone formation (such as teriparatide) are frequently transient, while side effects can be severe.^6^, ^7^ In a less invasive approach, Ca^2+^ and Mg^2+^ are given as dietary supplements, in the hope that this will elevate the intestinal concentration and thus, stimulate absorption.^5^ Side effects such as diarrhea and bloating are common, while the benefits are not undisputed.^8^, ^9^, ^10^ Thus, a large prospective study of 61 433 Swedish women over 19 years observed that intakes of Ca^2+^ near the top of the recommended range were paradoxically associated with an enhanced risk of hip fractures.^11^ Likewise, pooled results from various prospective cohort studies in different countries (170 991 women, 68 606 men) showed no reduction and a possible increased risk of hip fracture with calcium supplementation.^12^ In similar fashion, an excessive uptake of Mg^2+^ has been associated with negative outcomes.^2^ In this context, it should be noted that dietary Mg^2+^ can interfere with Ca^2+^ absorption and vice versa.^13^, ^14^, ^15^, ^16^ New therapeutic approaches are clearly desirable.

It has been known for some time that colonic absorption of divalent cations can be enhanced by adding “prebiotics” to the diet.^17^, ^18^, ^19^, ^20^, ^21^, ^22^, ^23^, ^24^, ^25^, ^26^, ^27^, ^28^, ^29^, ^30^, ^31^ Prebiotics are complex carbohydrates that are resistant to the enzymes of the small intestine and therefore enter the large intestine intact, where they are fermentatively degraded by resident microbials. In an era where populations are ageing, and vegan products are rapidly replacing milk as the major traditional source of dietary Ca^2+^ and Mg^2+^, a closer look at possible molecular mechanisms behind this effect appears merited.

## 2 HOMEOSTASIS OF Ca^2+^ AND Mg^2+^

### 2.1 Physiological functions of Ca^2+^ and Mg^2+^

Calcium is required for the maintenance of bone mineral density, muscle and cardiac contraction, synaptic transmission, blood coagulation, activation of enzymes, and cellular signaling, to name but a few functions.^13^, ^32^, ^33^ Serum calcium is normally maintained between 2.2 and 2.6 mmol L^−1^ by various hormones that regulate reabsorption by the kidney, release from bone, and buffering by plasma proteins. The net requirements for growth, pregnancy, lactation, and other needs of about 200 mg day^−1^ have to be covered by gastrointestinal absorption, some 70% of which has traditionally been supplied by milk and milk products in the Northern Hemisphere.

Serum Mg^2+^ is maintained between 0.7 and 1.1 mmol L^−1^, with recommended daily intake at roughly 400 mg.^33^ Both modern fertilization techniques and food processing have led to situations where Mg^2+^ intake is frequently inadequate.^34^ Lack of Mg^2+^ is associated with a host of health issues, including a correlation with diabetes mellitus type II, cardiovascular disease, and preeclampsia in pregnancy.^33^, ^35^ Furthermore, Mg^2+^ is a key stabilizing component of bone, where some 60% of the total amount in the body is stored.^2^, ^35^ Renal excretion rises with plasma Mg^2+^ levels, but the details of how Mg^2+^ homeostasis is regulated remain obscure, only that vitamin D is not involved.^13^, ^25^, ^33^

Both minerals are found abundantly in many types of food, but typically, less than 50% of the Mg^2+^ and less than 30% of the Ca^2+^ that is ingested enters the plasma. At about 70%, bioavailability is highest in milk and milk products for reasons that are not entirely clear.^13^, ^36^ Uptake is strongly influenced by the degree of mechanical and enzymatic digestion, the transit time, and the degree to which Ca^2+^ and Mg^2+^ are present in the ionized form.^13^, ^32^, ^37^, ^38^, ^39^

### 2.2 Intestinal absorption

The intestine is lined with a layer of cells that separate the lumen (or apical side) from the interstitial space (or basolateral side). Transport of ions across this epithelium can either occur through the cells (transcellular transport) or through the spaces between the cells (paracellular transport). Paracellular transport is always passive, with the direction of transport determined by the luminal concentration of the unbound (or “free”) ion and the transepithelial potential. Conversely, transcellular transport can also occur against electrochemical gradients, ensuring adequate uptake even when dietary content is low.

**FIGURE 1:**
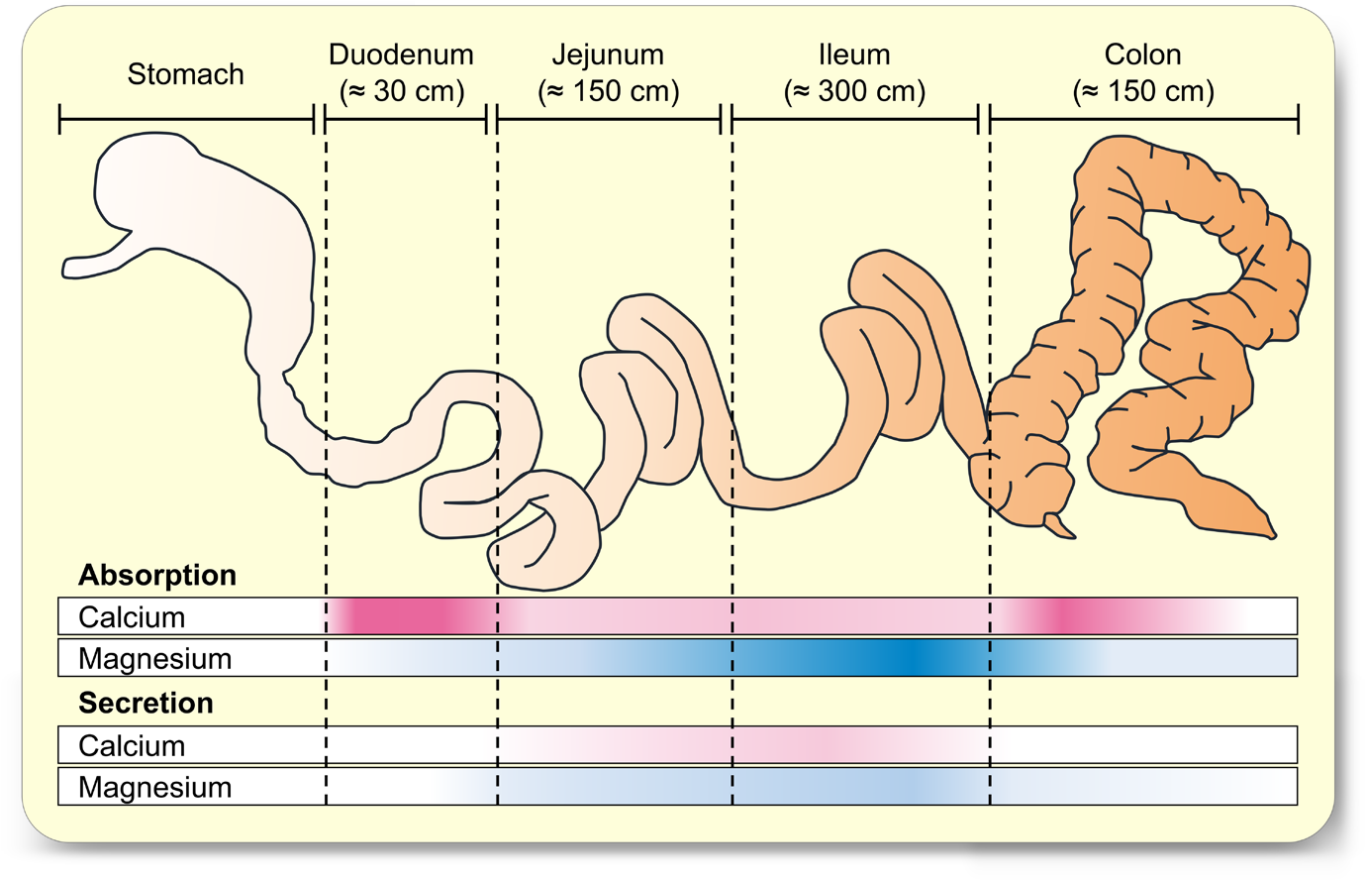
Intestinal transport of calcium and magnesium. Model of the human intestine, indicating the site of absorption and secretion of Ca^2+^ and Mg^2+^. Both the duodenum and the jejunum have a considerable capacity for active absorption of Ca^2+^ even when dietary intake is low. Due to its great length, the ileum is considered to be the major site for the absorption of both minerals. However, transport is passive, and a high luminal concentration is required to ensure net absorption. At low dietary intake, ileal secretion will predominate. Caecum and colon typically contribute to about 10%–30% of total Ca^2+^ uptake via active mechanisms and represent the major site of Mg^2+^ absorption in most species.

The absorptive capacity of the duodenum for Ca^2+^ is considerable, via vitamin D‐stimulated transcellular pathways that are energized by a basolateral Ca^2+^‐ATPase and Na^+^/Ca^2+^‐exchangers.^40^, ^41^, ^42^, ^43^ However, due to its short length, the duodenum usually only accounts for about 10% of net Ca^2+^ uptake, with another 10% absorbed by the jejunum (Figure 1). Accordingly, the long ileum has traditionally been considered to be the major locus of Ca^2+^ absorption in humans at high Ca^2+^ intakes, presumably via a passive paracellular pathway.^13^, ^33^, ^43^, ^44^, ^45^ Interestingly, however, high dietary intake of Ca^2+^ cannot compensate for the lack of vitamin D or its receptor, which argues against a major contribution of passive transport to calcium balance.^40^ Furthermore, at low dietary intake, the gradients across the ileum predict secretion instead of absorption. The precarious Ca^2+^ gradients across the small intestine sparked the search for active transcellular uptake mechanisms in the early 1960s^46^ and led to the identification of active calcium transport in the duodenum and jejunum, while transport across the ileum and colon is still poorly understood.^47^, ^48^

The colon has a pronounced capacity for absorption against an electrochemical gradient and typically contributes to another 10% of total Ca^2+^ uptake in humans.^23^ This amount may rise to about 30% on a fiber‐rich diet.^19^, ^20^, ^22^, ^31^, ^49^, ^50^, ^51^, ^52^, ^53^, ^54^ Notably, colonic uptake of Ca^2+^ and Mg^2+^ can become essential in patients in whom the small intestine has been partially removed.^55^, ^56^

In humans, uptake of Mg^2+^ by the duodenum plays a minor role, and absorption has classically been thought to occur primarily in the distal jejunum and ileum via passive mechanisms,^57^ although the colon is thought to play a major role in the fine‐tuning of Mg^2+^ absorption.^33^ Transport across the caecum and colon is active,^33^ and the hindgut has emerged as the major site of Mg^2+^ absorption in rats or pigs.^13^, ^58^ In ruminants, both the rumen and the colon predominate, while considerable secretion occurs along the ileum.^59^ As with Ca^2+^diets that enhance fermentational activity stimulate colonic Mg^2+^ absorption in various species, including humans.^17^, ^19^, ^20^, ^51^, ^52^, ^60^

### 2.3 Factors affecting the intestinal concentration of free Ca^2+^ and Mg^+2^

**FIGURE 2:**
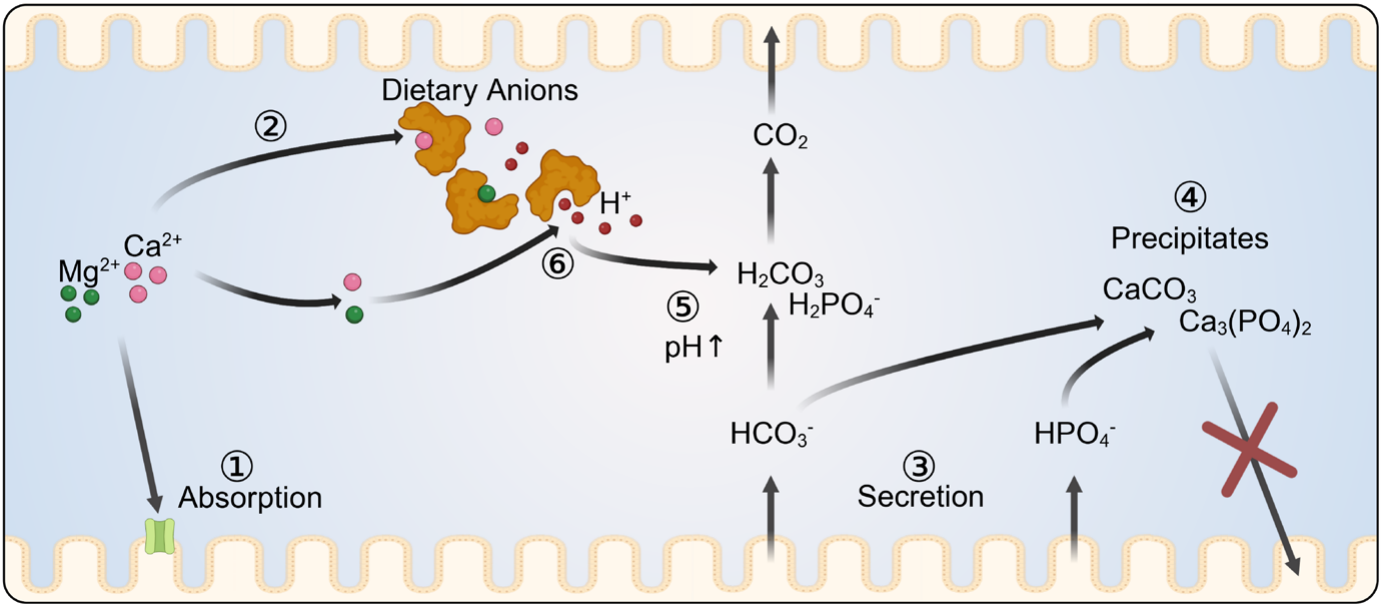
Buffering of Ca^2+^ and Mg^2+^ by luminal anions. Only the ionized forms of Ca^2+^ and Mg^2+^ are available for absorption (①). The concentrations of the free ions are reduced by dietary anions such as oxylates, tannins, and phytates, which form complexes with both cations (②). Furthermore, the endogenous secretion of HCO_3_^−^ and phosphates (③) leads to formation of insoluble carbonate and phosphsphate precipitates (④) and to an increase in pH (⑤). Protons are released from binding sites in chelating anions (⑥), which then bind Ca^2+^ and Mg^2+^ instead. Both dietary anions and the rate of endogenous secretion are thus major factors that limit the digestibility of Ca^2+^ and Mg^2+^ (created with BioRender.com).

Only the ionized or “free” form of Ca^2+^ and Mg^2+^ is available for absorption (Figure 2). Both endogenous secretion and the composition of the diet have large effects on the concentrations of the unbound ions, which may override any effect of dietary supplements.^1^ Thus, the luminal concentration of ionic Ca^2+^ and Mg^2+^ is strongly reduced by the presence of dietary oxalates, tannins, and phytates, which form complexes with both cations.^61^, ^62^ Such chelators are contained in large quantities in plant material, particularly in leafy vegetables. Furthermore, Ca^2+^ and Mg^2+^ form insoluble precipitates with phosphates and carbonates, both of which are endogenously secreted by intestinal epithelia and by the pancreatic gland in addition to being dietary components. On the other hand, low phosphorus intake can promote bone demineralization via a rise in serum parathyroid hormone (PTH).^63^ In animal nutrition, the amount of Ca^2+^ in the diet is therefore routinely balanced with the amount of dietary phosphate via defined ratios.^64^, ^65^ Studies in humans confirm that in order to improve calcium balance, it is essential to maintain a dietary calcium‐to‐phosphorus ratio within the range of 1.2:1 to 2:1, as is the case in milk.^36^

Paradoxically and despite absorptive processes, the concentration of free Ca^2+^ and Mg^2+^ in the gut lumen typically rises during transit through the intestine. This rise reflects the release of both minerals from complexes during digestion, concentration due to absorption of water, and paracellular secretion into the lumen from plasma. In a classical study of rats fed a diet with 0.18% w/w Ca^2+^,^66^ the concentration of ionized Ca^2+^ was found to be 0.19 mmol L^−1^ in the duodenum, 0.18 mmol L^−1^ in the jejunum, and 1.08 mmol L^−1^ in the ileum. All of these values are clearly lower than the concentration of Ca^2+^ in serum. In addition, many of the ileal transporters that mediate absorption of Na^+^ are electrogenic, resulting in an electrical gradient of >+6 mV, blood side positive.^38^, ^44^, ^67^ This electrochemical gradient clearly does not favor absorption via passive mechanisms and underscores the need for active, transcellular processes.

## 3 PREBIOTICS AND THE FERMENTATIONAL PROCESS

### 3.1 Prebiotics, prebiotic peptides, and probiotics

**FIGURE 3:**
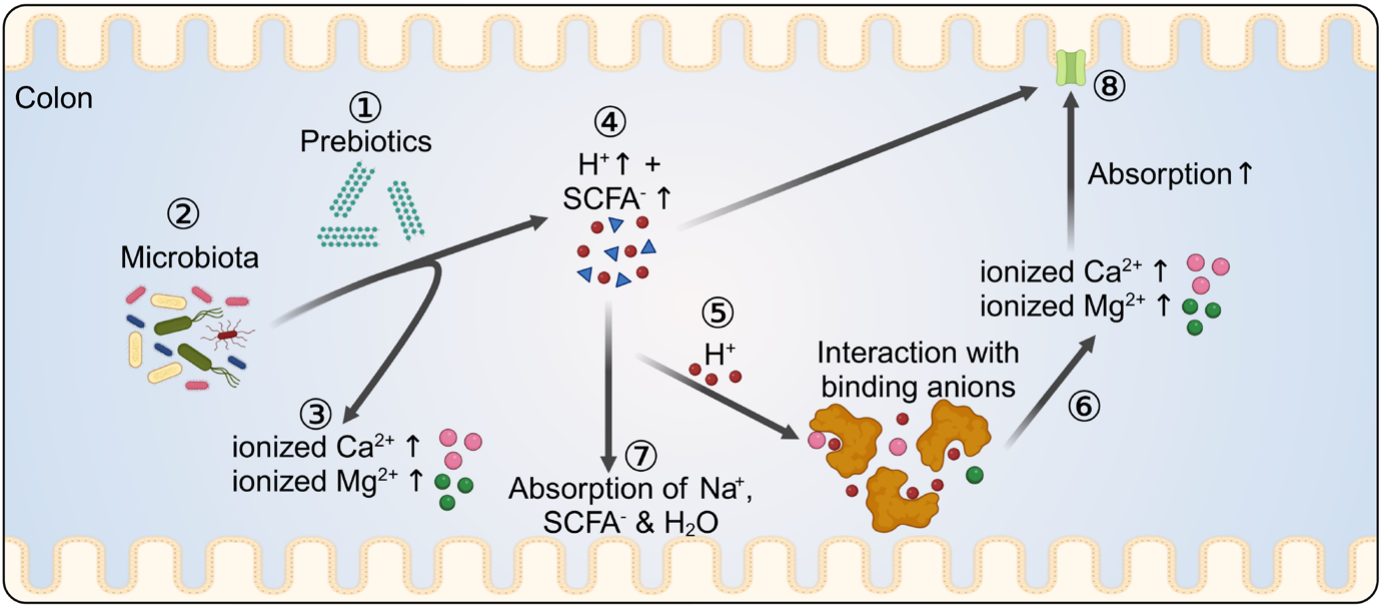
Effects of fermentation on the concentration of Ca^2+^ and Mg^2+^ in the hindgut. Prebiotics (①) are structural carbohydrates such as plant fiber, resistant starches, and inulin that cannot be digested by mammalian enzymes. However, microbiota (②) that colonize the gut can break up this undigested material, releasing the Ca^2+^ and Mg^2+^ contained within cells and complex molecules (③). In addition, the fermentation process leads to the formation of large quantities of short‐chain fatty acids (HSCFA) (④), such as acetic acid, propionic acid or butyric acid. Dissociation of HSCFA leads to the release of protons (⑤), which free Ca^2+^ and Mg^2+^ from binding sites in various dietary anions (⑥). SCFA also stimulate the absorption of Na^+^ and water from the gut, leading to a higher luminal concentration of Ca^2+^ and Mg^2+^ (⑦). Finally, there is reason to believe that fermentational activity also has direct effects on transport proteins, thus enhancing the absorption of Ca^2+^ and Mg^2+^ from the caecal or colonic lumen (⑧) (created with BioRender.com).

Fermentational activity can be enhanced by a variety of substrates that are resistant to the digestive processes of the small intestine and reach the hindgut, where they are broken down by resident microbials. Carbohydrate‐based compounds such as plant fiber, resistant starches, or inulin^68^ are designated as “prebiotics” and are primarily degraded to short‐chain fatty acids (SCFA) (Figure 3). Conversely, prebiotic peptides are synthetically produced resistant proteins that will additionally enhance the colonic production of branched‐chain fatty acids, phenols, indoles, hydrogen sulfide, amines, ammonia, and nitric oxide (NO).^69^, ^70^ Since most gut microbiota have a preference for carbohydrates over protein, one effect of adding prebiotics to the diet is to reduce the degradation of peptides.^69^, ^71^ Probiotics are viable microorganisms that are given with the intention of altering the microbiota in a manner that will be beneficial to the host, usually by shifting fermentation towards a higher production of SCFA.^49^, ^70^

Some 400 mmol day^−1^ of SCFA are normally produced in the human hindgut^72^, ^73^, ^74^ with luminal concentrations of SCFA between 90 and 150 mmol L^−1^. The concentration of branched‐chain fatty acids usually remains below 10 mmol L^−1^.^74^, ^75^, ^76^ SCFAs are absorbed to meet the energy needs not only of enteric cells but also of the individual as a whole. The low pK value of SCFA (~4.7) leads to pH values in the colon at around 6 in mice,^77^, ^78^ pigs^79^ and humans.^80^ Influx into the epithelial cells acidifies the cytosol, stimulating the absorption of Na^+^ via sodium‐proton exchange (NHE), followed by anions and water.

### 3.2 Effects of fermentation on the luminal concentration of free Ca^2+^ and Mg^2+^ in the large intestine

Fermentational processes in the hindgut lead to luminal concentrations of free Ca^2+^ and Mg^2+^ that are typically much higher than in either the small intestine or the plasma. There are several reasons for this. Firstly, the enzymatic activity of bacterial enzymes frees Ca^2+^ and Mg^2+^ from previously undigested material. Secondly, short‐chain fatty acids lower the luminal pH so that protons replace divalent cations in chelating anions (Figure 3). Thirdly, SCFA stimulate sodium‐proton exchange via NHE. The absorption of Na^+^, SCFA^−^, and water rises, leading to a further concentration of luminal Ca^2+^ and Mg^2+^ within the lumen. Accordingly, when rats were switched from a normal chow (~0.35% w/w Ca^2+^) to a diet high in a fermentable inulin–oligofructose mixture, caecal pH dropped from 6.8 to 5.3 with a concomitant increase in Ca^2+^ from 0.45 to 5.1 mmol L^−1^ ^31^

## 4 TRANSPORT MECHANISMS FOR Ca^2+^ AND Mg^2+^

### 4.1 Paracellular transport of Ca^2+^ and Mg^2+^

**FIGURE 4:**
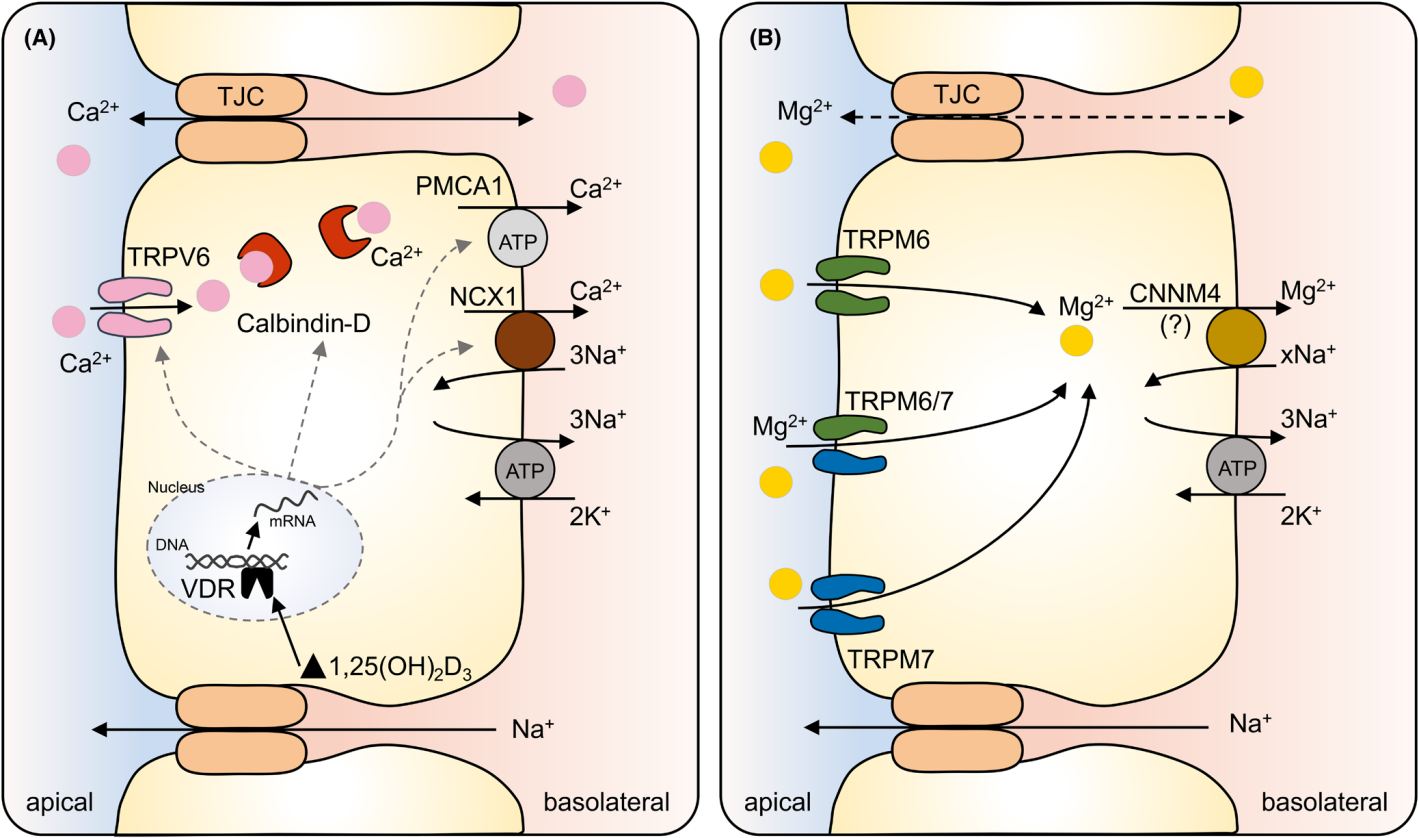
Transcellular and paracellular transport of Ca^2+^ and Mg^2+^. Intestinal epithelia consist of cells tightly adjoined to form a barrier between the gut lumen (apical side) and the interstitial space (basolateral side). The selectivity of the tight junction complexes (TJC) that interconnect the cells of the epithelium is modulated by various claudins, with the direction of transport depending on the electrochemical gradient. Alternatively, the ions may enter the cell via proteins within the apical cellular membrane and cross to the basolateral side, where efflux is directly or indirectly energized by ATP. Unlike the paracellular route, the transcellular pathway can mediate absorption even at low luminal concentrations. (A) Transport of Ca^2+^. Claudins 2, 12, and possibly also 15 contribute to the paracellular transport of this cation, in particular by the ileum. Unless the luminal concentration of ionic Ca^2+^ is high, secretion may exceed absorption. Transcellular uptake as found in the duodenum and jejunum consists of apical diffusion of Ca^2+^ into the cell via the Ca^2+^‐selective channel TRPV6, binding to calbindin, and basolateral exit primarily via the plasma membrane Ca^2+^‐ATPase (PMCA1), with a secondary active 3Na^+^/Ca^2+^‐exchanger (NCX1) playing a smaller role. The expression of all of these proteins is stimulated by calcitriol (1,25(OH)_2_D_3_). (B) The contribution of the tight junction to intestinal Mg^2+^ transport remains unclear. Transcellularly, TRPM6, TRPM7, and TRPM6/TRPM7 heteromeric channels mediate Mg^2+^ uptake into the cytosol. Extrusion is thought to occur in exchange for Na^+^ via cyclin and CBS domain divalent metal cation transport mediators (CNNM2 and/or CNNM4).

Epithelia consist of tightly adjoined cells that form a barrier between the lumen (or apical side) and the plasma (basolateral, serosal, or interstitial side) (Figure 4). A complex arrangement of proteins connects the adjacent cells of epithelia, either forming a tight barrier or allowing selective permeation of defined solutes through the space between adjacent cells (“paracellular pathway”).^81^ The phosphorylation, distribution, and expression levels of these proteins in general and of claudins in particular play a critical role in regulating the selectivity of the paracellular pathway.^82^, ^83^

#### 4.1.1 Tight junction proteins

In the kidney, claudins 16 (Cldn‐16) and 19 (Cldn‐19) selectively transport Ca^2+^ and Mg^2+^, but neither is expressed by the intestine.^84^ As will be discussed in more detail below, the poorly selective claudins 2, 12, and possibly also 15 have been associated with the intestinal transport of cations such as Na^+^, K^+^, and Ca^2+^.^85^, ^86^, ^87^

As mentioned, uptake of Ca^2+^ and Mg^2+^ by the ileum is thought to occur primarily via the paracellular pathway.^33^ However, the usefulness of this pathway for cation absorption is debatable, since the gradients are frequently insufficient and predict secretion. Note that the ileum can maintain steep gradients for chloride,^88^ arguing against the classical hypothesis of simple paracellular “pores” mediating flux down the electrochemical gradient.

#### 4.1.2 Solvent drag theory

The problem of an insufficient driving force for paracellular uptake has long vexed researchers.^89^ In theory, at least, paracellular transport processes of individual ions against their electrochemical gradients can occur via flux coupling. In the classical model, an osmotic gradient is generated via transcellular active mechanisms and then drives the uptake of water, flushing other ions along through large, non‐selective paracellular pores. Solvent drag theory was thus initially utilized to explain the uptake of sugars and amino acids from the intestine. This theory was replaced after the discovery of active, sodium‐coupled cotransport and by the finding that in healthy, intact tissues, tight junction proteins efficiently prevent the non‐selective fluxes of large solutes.

It is becoming increasingly clear that classical solvent drag theory may have to be reassessed.^90^ Thus, only one claudin, claudin‐2, is permeable to water, and this claudin typically only plays a role in pathological situations with enhanced secretion into the intestine.^91^, ^92^ In the healthy gut, claudin‐15 is expressed, but this claudin is impermeable to water.^92^ Instead, at least in mice, claudin‐15 is essential for the secretion of Na^+^ from plasma into the gut lumen for the uptake of nutrients via Na^+^‐cotransporters.^93^ The electrochemical and osmotic gradients thus drive Na^+^ into the lumen, and a pore with solvent drag would therefore flush Ca^2+^ and Mg^2+^ into the intestinal lumen and not out of it. Indeed, it has been suggested that one effect of calcitriol may be to downregulate Cldn‐2 in order to reduce secretion of Ca^2+^ into the intestinal lumen.^87^, ^94^, ^95^ In conjunction, in most scenarios, transcellular uptake mechanisms are required for uptake.

### 4.2 Transcellular transport of Ca^2+^

It has been established without reasonable doubt that transport of Ca^2+^ across the duodenum and jejunum is predominantly transcellular. Transport involves transient receptor potential vanilloid type 6 (TRPV6), which mediates the apical entry of Ca^2+^ into enterocytes of these intestinal segments.^43^, ^96^, ^97^ (Figure 4A).

Studies under the controlled condition of the Ussing chamber suggest that despite the favorable gradients, absorption of Ca^2+^ and Mg^2+^ across the hindgut also primarily occurs via active transcellular pathways.^33^, ^98^, ^99^ The rat caecum, in particular, has a very pronounced capacity for active calcium transport that exceeds even that found in the duodenum.^100^, ^101^

The Ca^2+^‐selective channel TRPV6 is strongly expressed by the duodenum, caecum, and colon, with smaller amounts found in the jejunum but an almost total absence in the ileum. Conversely, expression of its close relative TRPV5 is largely limited to the distal convoluted and connecting tubules of the kidney, while any expression by the intestine is very low.^102^, ^103^, ^104^, ^105^, ^106^

After apical uptake, calcium‐binding proteins such as calbindin‐D_9k_ keep the cytosolic concentration minimal (~100 nmol L^−1^), important for cell viability and ensuring a steep electrochemical gradient for apical influx. The rapid transport to the basolateral side involves mechanisms other than simple diffusion. According to one model, the Ca^2+^‐calbindin‐D_9k_ complex attaches to the apical end of the endoplasmic reticulum (ER), where Ca^2+^ is taken up by SERCA pumps. This induces a signaling cascade that allows an equivalent amount of Ca^2+^ to be released from the basolateral end of the ER via Ca^2+^‐release channels.^39^, ^42^, ^107^ Export from the cytosol into the interstitial space is energized via the plasma membrane Ca^2+^‐ATPase 1b (PMCA1b), with a smaller role played by the sodium‐calcium exchanger (NCX1).^39^, ^108^, ^109^, ^110^ All of these mechanisms are upregulated by high calcium demand or a low calcium diet.^42^ Most of this effect is mediated by the active form of vitamin D (calcitriol, 1,25‐Dihydroxy‐Vitamin D, or 1,25(OH)_2_D3) via the vitamin D receptor (VDR).^39^, ^43^, ^44^, ^111^, ^112^, ^113^, ^114^

TRPV6 knockout animals show impaired calcium homeostasis but are viable even when dietary Ca^2+^ intake is restricted.^47^, ^102^, ^104^, ^113^, ^115^, ^116^, ^117^ It has therefore been suggested that TRPV6 may not be the only intestinal Ca^2+^ channel.^41^, ^47^, ^118^ A logical additional candidate gene is TRPV5, but studies confirmed that even in TRPV6 KO mice, mRNA encoding for TRPV5 was below the detection level throughout the intestine.^112^ In another study of mice expressing a non‐functional mutant, duodenal TRPV5 expression increased very slightly to a mere 1% of the level of TRPV6 in the WT mice.^117^

A role for Ca_V_1.3 in the apical uptake of Ca^2+^ from the ileum has been suggested but remains controversial.^23^, ^119^ Given that Ca_V_1.3 is inhibited when external pH drops below 6.7,^120^ any role in the uptake of Ca^2+^ from the fermentational parts of the gut is likely to be marginal. In this context, it is interesting to note that the intestine also expresses a large number of non‐selective members of the TRP channel family, the role of which is currently poorly understood.^121^

#### 4.2.1 Properties of TRPV6

Structurally, the transmembrane domain of the apical Ca^2+^ channel TRPV6 resembles the general architecture of voltage‐gated Na^+^ and K^+^ channels, although in TRPV6, the S4 helix does not convey voltage sensitivity. In the presence of extracellular Ca^2+^, although not in its absence, TRPV6 is almost completely impermeable to the influx of monovalent cations.^122^ The properties of TRPV6 and the renal channel TRPV5 are remarkably similar, since the exon size in the coding region is completely conserved, suggesting evolutionary descent from a single ancestral gene.^114^, ^123^ An elevation of intracellular Ca^2+^ blocks both channels via interaction with the TM2–TM3 loop, albeit with differing kinetics in the two channels.^122^, ^124^, ^125^ In addition to cytosolic buffering (in particular via calbindin‐D_9K_), this mechanism may help to prevent a toxic elevation of cytosolic Ca^2+^.

Interestingly, protons block both TRPV5 and TRPV6 via the binding of intra‐ and extracellular protons to a lysine 607 amino acid residue in the pore region.^126^, ^127^, ^128^, ^129^, ^130^ Since fermentational processes in the hindgut have an acidifying effect on both the lumen and the cytosol, a reduction of Ca^2+^ transport via TRPV6 is expected when fermentation increases, in contrast to what is observed, arguing for an alternate pathway in this part of the gut.

Mg^2+^ does not permeate TRPV6 but blocks the conductance to Na^+^ in a voltage‐dependent manner from both the extra‐ and the intracellular side. Mediated by a single aspartate (Asp^541^) that is located in the selectivity filter,^131^ the block of TRPV6 by Mg^2+^ may explain the negative effect of high dietary Mg^2+^ on Ca^2+^ uptake.^13^

### 4.3 Transcellular transport of Mg^2+^

A subgroup of TRP channels, namely TRPM1, TRPM3, TRPM6, and TRPM7, consists of proteins that are highly permeable to divalent cations such as Mg^2+^, Ca^2+^, and Zn^2+^.^132^ While mutations of TRPM1 lead to impairment of night vision, loss of TRPM3 is associated with epilepsy and intellectual disability. Neither of the two leads to an apparent disturbance of systemic Mg^2+^ or Ca^2+^ homeostasis.

In contrast, the Mg^2+^‐permeable transient receptor potential cation channel subfamily M member 6 (TRPM6) was identified as a candidate gene for Mg^2+^ uptake after screening families with autosomal recessive hypomagnesemia.^133^, ^134^, ^135^, ^136^ *Homozygous* mutations of TRPM6 cause severe hypomagnesemia, and hypocalcemia in newborns with seizures and tetany. All symptoms, including hypocalcemia are restored by supplementation with Mg^2+^, highlighting the importance of the channel‐mediated transcellular pathway for Mg^2+^ homeostasis.^133^, ^134^, ^137^ Studies in knockout mice confirm that TRPM6 plays a central role in Mg^2+^ balance that cannot be compensated for by any other channel.^138^ This clearly challenges the classical notion that uptake of Mg^2+^ occurs primarily via diffusion across the paracellular pathway of the ileum.^33^, ^57^ Instead, mounting evidence suggests that, as in pigs, rodents, or cattle, a considerable proportion of Mg^2+^ uptake in mice and in humans occurs in the hindgut via a transcellular route.^35^ Both caecum and colon express TRPM6 in the apical membrane (Figure 4B).^33^, ^59^, ^121^, ^135^, ^139^, ^140^

A second Mg^2+^‐permeable channel, TRPM7, is ubiquitously expressed by tissues throughout the body and is considered an essential housekeeping gene in the service of cellular Mg^2+^ homeostasis.^141^, ^142^, ^143^ Thus, TRPM7 opens when intracellular ionized Mg^2+^ concentration drops below the normal ~1 mmol L^−1^.^144^ Mice homozygous for null alleles of TRPM7 die in the embryonic stage.^132^ In humans, certain variants of TPRM7 with decreased channel expression or activity are viable, although hypomagnesemia is observed despite normal expression of TRPM6.^145^

These observations and a variety of in vitro experiments support the notion that TRPM6/7 heteromers are essential for apical Mg^2+^ uptake.^140^, ^146^ Unlike the homomeric channels, these heteromers are not as susceptible to inhibition by rising levels of intracellular Mg–ATP and can thus mediate the uptake of large amounts of Mg^2+^ in transporting epithelia.^132^, ^138^ This may explain why expression of TPRM7 cannot compensate for loss of TRPM6 and vice versa.

Expression patterns of TRPM6/TRPM7 appear to vary. In one study of mice, any expression of TRPM6 by the duodenum or jejunum was marginal, although interestingly, a slightly higher expression was observed in the ileum.^139^ A study of rats saw expression increase from duodenum to colon.^78^ Conversely, another study of rats on the level of the protein showed that membrane and cytosolic expression of TRPM6 was highest in the duodenum and declined thereafter.^147^ In pigs, a pronounced peak was found in the jejunum that exceeded the other two maxima in the caecum and colon, with marginal expression elsewhere.^79^

Basolateral efflux probably involves cyclin M4 (Cyclin And CBS Domain Divalent Metal Cation Transport Mediator 4, CNNM4), which most likely functions as a Na^+^/Mg^2+^‐exchanger with unclear stoichiometry.^33^ However, human patients with CNNM4 mutations do not suffer from hypomagnesemia, although impaired retinal function and tooth mineralization are observed.^148^ Instead, mutation of the close relative CNNM2 causes hypomagnesemia and seizures^149^ and probably also plays a certain role in intestinal uptake.^150^ It is not entirely clear if CNNM2 is a Mg^2+^ transporter or an associated protein.^151^ Likewise, MAGT1, which was originally thought to mediate magnesium transport, appears to indirectly influence magnesium homeostasis by facilitating N‐linked protein glycosylation.^152^ In the kidney, solute carrier family 41 members 1, 2, and 3 are thought to mediate basolateral renal Mg^2+^ transport via exchange with Na^+^ ^35^, ^136^, ^153^ with a recent study in mice demonstrating a role for SLC41A3, but not for SLC41A1.^154^ However, possibly, the primary role of SLC41A3 is to mediate mitochondrial efflux of Mg^2+^ while the role for intestinal uptake remains unclear.^35^, ^155^, ^156^ A number of other candidate genes have been proposed to mediate Mg^2+^ transport, but their participation remains speculative.^136^

Due to binding to a variety of anions,^33^ intracellular ionized Mg^2+^ levels lie roughly between 0.2 and 1 mmol L^−1^. The driving force for the apical uptake of Mg^2+^ is mostly due to the negative membrane potential. Depolarization of the apical membrane—e. g., by K^+^—can thus interfere strongly with the absorption of Mg^2+^. In ruminants, the negative effects of K^+^ on Mg^2+^absorption can lead to death from hypomagnesemia .^157^, ^158^

#### 4.3.1 Properties of TPRM6 and TRPM7

TRPM6 shows considerable structural similarity to other members of the TRP channel family and 50% homology with TRPM7. TRPM6 and TRPM7 assemble as homomeric or heteromeric tetramers, forming transmembrane channels with relevance for the uptake not just of Mg^2+^, but also of other divalent cations such as Ca^2+^ and Zn^2+^ or even Cd^2+^.^144^, ^159^, ^160^ Notably, divalent cations compete with each other for permeation through TRPM6, so that permeation of trace metals is most likely blocked by the more abundant species, Ca^2+^ and Mg^2+^.^160^ Furthermore, it appears possible that high amounts of Ca^2+^ will interfere with the uptake of Mg^2+^.

Of all mammalian ion channels studied so far, only TRPM6 and TRPM7 are fused to a kinase domain, allowing them to phosphorylate downstream targets. suggesting functions beyond epithelial transport.^159^, ^161^ TRPM7 is thought to play important roles in various pathways that trigger cell growth, differentiation, apoptosis, or inflammation, both by functioning as an enzyme and by mediating the influx of Mg^2+^ that is central to the signaling cascade of this tyrosine kinase and others.^144^ Furthermore, various hormones that trigger cellular growth, such as estrogen or epidermal growth factor, are known to upregulate the expression of TRPM6 and TRPM7.^139^, ^144^ High expression levels of TRPM7 are linked to tumor growth and associated with a poor prognosis.^162^, ^163^ Interestingly, cancer patients that are treated with the EGFR antagonist cetuximab frequently develop severe hypomagnesemia that is associated with a decreased expression of TRPM6 and possibly TRPM7.^144^, ^164^

Both TRPM6 and TRPM7 respond to changes in pH. Protons interact with a negatively charged head group of an intracellular gating site, leading to a decrease in the open probability.^165^, ^166^, ^167^ Furthermore, a negatively charged side chain of a glutamate residue (E1024 for TRPM6 and E1047 for TRPM7) that is located in the pore‐forming loop of the channels forms a cation‐binding site that can discriminate between monovalent and divalent cations.^146^, ^159^ At neutral pH, Ca^2+^ and Mg^2+^ bind to the pore region so that the hydration shell is removed and both ions can permeate, while the transport of monovalent ions is blocked. Conversely, at low external pH, this negative binding site is saturated by protons, and the channel becomes permeable to monovalent cations.^146^, ^168^ This can lead to confusion. External protons do not enhance uptake of Mg^2+^ or Ca^2+^ through TRPM6 and TRPM7 as is sometimes suggested but instead enhance monovalent currents through the channel.^146^, ^160^, ^168^ Conversely, it has been shown that an influx of weak acids such as acetic or propionic acid inhibits currents through TRPM7, while weak bases such as ammonia increase the channel conductance, with similar effects observed after direct alteration of the internal pH in the range between 8.4 and 5.6, as found in fermentative organs.^165^, ^166^ Notably, SCFA can also be expected to increase ATP production, which inhibits TRPM6.^169^ In summary, TRPM6/TRPM7 do not appear as likely candidate genes explaining the increase in Mg^2+^ uptake in response to increased colonic fermentation with the production of SCFA and protons.

## 5 THE EFFECT OF PREBIOTICS ON INTESTINAL ABSORPTION OF Ca^2+^ AND Mg^2+^

A solid body of literature, both in vitro and in vivo, suggests that prebiotics stimulate the absorption not only of Ca^2+^, but also of Mg^2+^ and other trace minerals from the intestines of various species, including rodents, pigs, cattle, and humans.^17^, ^18^, ^19^, ^20^, ^21^, ^22^, ^23^, ^24^, ^25^, ^26^, ^170^ An overview of the effects of various prebiotics on the absorption of Ca^2+^ and Mg^2+^ can be obtained in several excellent reviews of the topic.^23^, ^26^, ^49^

Improvement of Ca^2+^ and Mg^2+^ absorption from the intestine and/or increase in bone mineral density has been reported repeatedly in studies of rats or mice that were fed complex carbohydrates to stimulate fermentational activity.^171^, ^172^, ^173^, ^174^, ^175^ To give some examples, studies of rats in vivo demonstrated that feeding fructooligosaccharides stimulated absorption of Ca^2+^ and Mg^2+^ across the hindgut.^176^, ^177^ Also in rats, feeding galactooligosaccharides increased net uptake and retention of Ca^2+^ and Mg^2+^, femur and tibia breaking strength, and distal femur total and trabecular volumetric bone mineral density.^178^ In a comparative study of ovariectomized rats, those fed polydextrose or two different types of inulin had significantly higher absorption and retention of Ca^2+^ and Mg^2+^ than a control group or a group receiving daily estradiol injections.^51^ In mice treated with the proton‐pump inhibitor omeprazole, serum Ca^2+^ levels could be restored by co‐feeding with inulin, although Mg^2+^ levels remained low.^22^

In postmenopausal women, dietary oligofructose‐enriched inulin improved mineral absorption (measured via dual stable tracer isotopes of Ca^2+^ and Mg^2+^) and bone formation (via bone turnover markers) in patients with lower initial spine bone mineral density.^20^ A study of adolescent girls found that a diet rich in soluble corn fiber increased absorption of Ca^2+^ as measured by stable isotope (^44^Ca^2+^ and ^43^Ca^2+^) enrichment in pooled urine as measured by mass spectrometry.^179^ Conversely, a study in adolescent girls with low Ca^2+^ intake found stimulatory effects of short‐chain fructooligosaccharides on the absorption of Mg^2+^ alone without impact on Ca^2+^ uptake.^180^ In human patients suffering from hypomagnesemia, inulin significantly increased serum Mg^2+^ levels and alleviated clinical symptoms,^52^ although other studies in humans failed to find significant effects.^18^, ^180^ The variability of the findings may relate to the difficulties in controlling experimental conditions, in obtaining enough human participants, and in differences in the response of individuals, possibly also reflecting the pre‐trial mineral status and the microbiome of the individual.^181^ Furthermore, evidence has emerged that certain individuals respond to a high fiber diet with general increases in basal immune activation with possible detrimental effects on pre‐existing conditions, resulting in defects in intestinal barrier function.^182^

## 6 PREBIOTICS AND THE ENTERO‐OSSEUS SIGNALING AXIS

Prebiotics may also lead to hormonal stimulation of bone formation via the gut–bone signaling axis.^68^ The complexity of the gut–bone interactions is immense and poorly understood, but a brief overview of some emerging topics will be attempted.^183^, ^184^

Levels of vitamin D are not affected by prebiotics, and their positive effects partially persist even when the supply of vitamin D is insufficient.^185^ Intriguingly, however, a recent study has shown that the short‐chain fatty acids butyrate and propionate upregulate VDRs in cultured hepatocytes.^186^ If confirmed in vivo, prebiotics might stimulate the hepatic conversion of cholecalciferol to 25‐hydroxyvitamin D_3_ and finally, to the active form calcitriol with stimulation of Ca^2+^ absorption by the kidney and the gut and stimulation of bone formation. It should be noted that effects on Mg^2+^ uptake are not to be expected.

One study has suggested that the short‐chain fatty acids propionate and butyrate directly affect bone formation by suppressing osteoclasts.^183^

Despite some degradation of H_2_S within the colonic epithelium and the liver, studies in mice suggest that a considerable portion of circulating H_2_S results from the bacterial degradation of protein and other sulfur‐containing substrates.^69^, ^187^, ^188^ In bone, H_2_S triggers the differentiation of both osteoclasts and osteoblasts.^189^, ^190^ Intriguingly, in a study of mutant mice with low endogenous H_2_S due to a mutation of certain H_2_S‐producing enzymes, osteoporotic symptoms could be alleviated by non‐toxic H_2_S donors.^190^ However, and as mentioned, carbohydrate‐based prebiotics will normally suppress the formation of H_2_S so that this signalling molecule cannot explain their effects on bone formation.^71^

**FIGURE 5:**
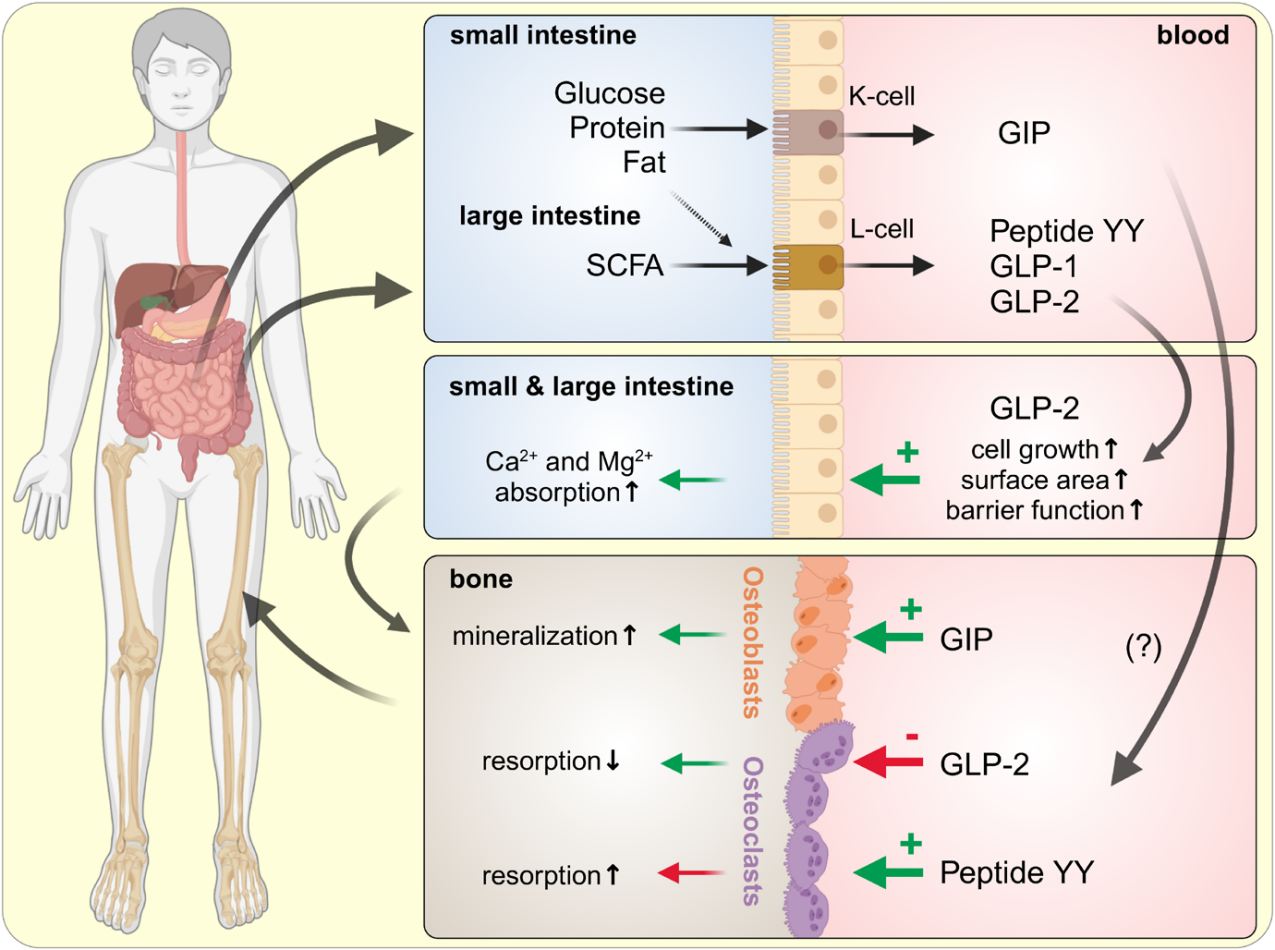
Some effects of prebiotics on the entero‐osseous signaling axis. The enteroendocrine hormones GIP, GLP‐1, and GLP‐2 have well‐documented effects on insulin secretion, energy homeostasis, and satiety signaling. Recent research suggests possible roles in bone turnover. In the upper small intestine, glucose, protein, and in particular fat trigger the secretion of GIP from K‐cells, while a smaller number of L‐cells secrete GLP‐1 and GLP‐2. However, most L‐cells are found in the hindgut, where SCFA trigger the release of GLP1, GLP2, and peptide YY. GLP‐2 has well‐documented effects on enhancing the surface area and transporting capacity of gastrointestinal epithelia. Furthermore, effects of gastrointestinal hormones on the removal of bone by osteoclasts or formation of new bone by osteoblasts have been reported. The field is in flux, and more clinical data are required (for details, see text) (created with BioRender.com).

The enteroendocrine hormones GIP, GLP‐1, and GLP‐2 have received much attention due to their effects on insulin secretion, energy homeostasis, and satiety signaling, but interestingly, they have also been implicated in preventing osteoporosis. This can involve enhancing the formation of new bone tissue by osteoblasts and stimulating the removal of aged bone by osteoclasts, or attenuating excess osteoclast activity that might degrade bone strength (Figure 5).^191^, ^192^

Glucose, protein, and in particular, fat trigger the secretion of the incretin GIP from “K‐cells,” which are primarily found in the upper small intestine.^193^ GIP typically stimulates insulin secretion by the pancreas, but in addition, GIP receptors are expressed by osteoblasts, and both anabolic and anti‐catabolic actions that enhance bone quality have been reported in rodents and humans.^191^, ^194^, ^195^

Three other peptide hormones, GLP‐1, GLP‐2, and peptide YY, are produced by “L‐cells”. Interestingly, the number of L‐cells is low in the proximal intestine and rises distally, with the highest number found in the caecum, colon, and rectum.^193^ Since glucose cannot be found in these segments, SCFA are the major triggers leading to endocrine secretion of GLP‐1 and peptide YY from L‐cells^196^, ^197^ or of serotonin from EC cells.^198^ Encouraging data from in vitro models and studies in rodents have generated some excitement, but the rodent response to GLP‐1 appears to be mediated by increased secretion of calcitonin, a calcitropic hormone without measurable impact in humans. Any effects of GLP‐1 or GLP‐1 agonists on human bone homeostasis are debatable.^191^, ^199^, ^200^ Peptide YY, or gut‐derived serotonin, may affect bone turnover by stimulatory effects on osteoclasts.^184^, ^201^, ^202^ More clinical studies are required.

Evidence for the positive effects of GLP‐2 on bone health in humans is more robust.^194^, ^203^, ^204^ A direct inhibitory effect on bone resorption by osteoclasts has been suggested,^203^ or an inhibition of PTH secretion via GLP‐2 receptors expressed by the parathyroid gland.^205^ Classical and well‐documented effects of GLP‐2 include the proliferation of the absorptive surface of intestinal epithelia with a higher expression and activity of epithelial brush‐border nutrient transporters, leading to higher rates of mineral absorption.^206^

An ample literature supports the stimulatory effects of SCFA on hindgut proliferation^207^ with an increase in surface area for absorption. To name but one example, in a study of rats fed a prebiotic, caecal surface area increased by a factor of two, doubling the total transport capacity of this organ.^31^

## 7 POSSIBLE MECHANISMS FOR THE STIMULATORY EFFECTS OF PREBIOTICS ON THE ABSORPTION OF Ca^2+^ AND Mg^2+^ FROM THE HINDGUT

One study has suggested that prebiotics directly interact with phytic acid and other anions in the small intestine, freeing Ca^2+^ and Mg^2+^ from binding.^208^ However, a clear majority of studies suggest that the stimulatory effects of prebiotics follow their degradation in the hindgut.^23^, ^26^, ^31^, ^49^ Thus, adding prebiotics to the diet of ileostomy patients lacking caecum and colon typically has no impact on mineral absorption.^209^ Furthermore, a tracer study using ^45^Ca^2+^ in adolescents found that consuming inulin‐type fructans increased the absorption of Ca^2+^ from the gut, with 70% occurring >7 h after dosing.^50^ The timeline argues for effects following an entry into the large intestine.

One possible explanation is that fermentational processes release large quantities of protons that can displace divalent cations from chelating anions, enhancing luminal concentration. However, rectal infusion of solutions containing Na‐acetate and/or Na‐propionate (56.3 mmol L^−1^) with pH buffered to 7.0 stimulated the absorption of Ca^2+^ from the rectum, sigmoid colon, and colon descendens of healthy human volunteers in vivo with significant effects observed after as little as 10 min.^28^, ^30^ Conversely, an infusion of an acidic control NaCl solution (pH 4.9) did not show these effects. These classical observations argue for direct effects of prebiotics or the SCFA derived from them on transport proteins or on the proteins lining the paracellular pathway.

### 7.1 Possible effects of prebiotics on the paracellular pathway

In the caecum and colon, the gradients are usually sufficient to drive passive uptake, and an attractive hypothesis is that prebiotics stimulate paracellular uptake mechanisms. In Ussing chamber experiments of the jejunum, ileum, caecum and colon of rats, mucosal addition of high concentrations (up to 100 mmol L^−1^) of indigestible saccharides stimulated Ca^2+^ uptake.^210^ In these experiments, a Ca^2+^ gradient (10 mmol L^−1^ mucosal/1.25 mmol L^−1^ serosal) was present. In control experiments, equimolar amounts of glycerol were added, which did not have a stimulatory effect. However, note that glycerol can enter cells via aquaporins.^211^ The simplest explanation for the observations is that the addition of saccharides led to a hyperosmolar situation with cell shrinkage and opening of the paracellular pathway, while the control solution was hyperosmolar but isotonic, so that cells did not shrink and no effect was observed. However, in a second study in which tonicity was maintained throughout, various non‐digestible saccharides (50–100 mmol L^−1^) also opened a paracellular calcium transport pathway in a colonic cell culture model (Caco‐2).^212^ In all of these scenarios, the concentrations of unfermented prebiotics used appear to be very high indeed, and fermentation would yield many times the concentration of SCFA found physiologically. The relevance of these observations for the situation in vivo is thus debatable.

Another study of Caco‐2 cells demonstrated that treatment with butyrate (2 mmol L^−1^) resulted in a tightening of barrier function as measured via transepithelial resistance, most likely via a reorganization of preexisting tight junction proteins.^213^ At higher levels of butyrate (8 mmol L^−1^), apoptosis occurred in this cell culture model. Increasing levels of apoptosis may explain why in another study of Caco‐2 cells, Na‐butyrate (2–8 mmol L^−1^) led to a concentration‐dependent reduction in ATP production and Mg^2+^ uptake.^214^

Note that in vivo, a thick layer of mucous protects the cells of the epithelium from excessive influx of SCFA,^215^ which is a possible reason why luminal concentrations of 8 mmol L^−1^ of butyrate are not only tolerated but even have well‐documented barrier‐enhancing effects on the intact tissue.^216^, ^217^ A tight barrier is required so that the colon can absorb water and electrolytes against steep gradients. On the other hand, unphysiologically high rates of fermentation with very high levels of SCFA can lead to barrier breakdown with an influx of colonic metabolites into the bloodstream.^218^

### 7.2 Effects of SCFA on the transcellular pathway

The rapid stimulation of Ca^2+^ from the hindgut of humans by SCFA, as mentioned above^28^, ^30^ is supported by studies in animal models. In a study of rat distal caecum and colon using an in vivo luminal perfusion technique, SCFA stimulated Ca^2+^, Mg^+^ and K^+^ absorption by the distal colon within 30 min.^24^, ^219^, ^220^ In these studies, physiological buffer solutions containing either butyrate, acetate (60 mmol L^−1^) or a physiological mixture of SCFA (60 mmol L^−1^ acetate, 20 mmol L^−1^ propionate, 10 mmol L^−1^ butyrate) were used. Notably, the mucosal solutions only contained 1 mmol L^−1^ Ca^2+^. Given that the potential across the colonic epithelium is blood‐side positive, this suggests that an active, transcellular transport mechanism must have been involved that responded to changes in the SCFA concentration. From various observations, the authors concluded that SCFA derived from the fermentation of carbohydrates in the large intestine provide cytosolic protons for the stimulation of apical Ca^2+^/H^+^ and Mg^2+^/H^+^‐exchangers. Such exchangers, however, have yet to be found, and an alternative explanation of the findings is that intracellular acidification stimulates influx through a divalent permeable ion channel.

Some interesting conclusions can be drawn from studies of ruminants, which have a particularly high requirement for Ca^2+^ and Mg^2+^ due to the large quantities that are secreted with milk.^59^, ^157^, ^221^, ^222^, ^223^, ^224^ In these animals, over 50% of total Ca^2+^ and Mg^2+^ absorption occurs across the rumen, a large fermentation chamber that precedes the stomach and the intestine.^59^, ^157^, ^221^, ^224^, ^225^ Effects of SCFA on the uptake of Ca^2+^ and Mg^2+^ in the rumen have been extensively studied in different laboratories, both in sheep in vivo and across isolated epithelia in Ussing chambers ex vivo.^222^, ^226^, ^227^, ^228^, ^229^, ^230^ In the case of the Ussing chamber studies, conditions were tightly controlled with the absence of an electrochemical gradient for Ca^2+^ and Mg^2+^, defined values of pH, and no changes in osmolarity. Stimulatory effects of SCFA rose with chain length and luminal proton concentration. The rapid onset of the stimulatory effects within an hour suggests that at least part of the effect was due to a direct interaction of protons or SCFA with preexisting transport proteins rather than changes in protein expression. Similar conclusions have also been drawn from studies on rats or mice.^31^, ^231^

Interestingly, blocking the sodium‐proton exchanger (NHE) via amiloride enhanced Ca^2+^ transport across the rumen, suggesting a stimulatory effect of intracellular acidification on Ca^2+^ uptake.^227^ This argues against an involvement of either TRPV5 or TRPV6, channels that are inhibited by acidification.^126^, ^127^, ^128^, ^129^, ^130^ Indeed, any expression of TRPV6 (or TRPV5) by the rumen is marginal,^170^, ^232^, ^233^, ^234^ and Ca^2+^ uptake in cattle cannot be significantly enhanced by calcitriol.^221^, ^224^, ^233^, ^235^

Unfortunately, very few Ussing chamber studies of rat caecum and colon exist, and these were performed in the presence of a gradient for Ca^2+^ (10 mmol L^−1^ mucosal, 1.25 mmol L^−1^ serosal).^31^, ^236^ A strong stimulatory effect of SCFA was observed, which might, however, be due to a stimulation of either transcellular or paracellular transport. Interestingly, a reduction of mucosal pH (from 7.4 to 6.0) had no effect in these experiments.^31^ Prefeeding rats with a diet containing a prebiotic also had no effect on Ca^2+^ flux in Ussing chambers.^31^

### 7.3 Effects of hydrogen sulfide (H_2_S) and NO

Colonic H_2_S is produced primarily by gut bacteria, while NO levels are determined both by production via the colonic mucosa and by anaerobic species that denitrify nitrates.^237^ Dietary strategies attempting to raise the colonic concentration of H_2_S and NO must provide additional nitrogen and sulfur, e.g., by adding prebiotic peptides to the diet.^69^, ^71^ Conversely, adding “classical” carbohydrate‐based prebiotics such as fiber will reduce the production of these metabolites.^71^ Despite this caveat, a closer look at these gaseous transmitters is merited.

Countless proteins contain cysteine (Cys) residues or SH groups that can be converted to a persulfide or –SSH group by H_2_S, leading to conformational changes with functional consequences. Alternatively, nitrosylation by NO can occur.^190^, ^238^, ^239^ Molecular targets include K_ATP_ channels, BK_Ca_ channels, proline‐rich kinase 2, and the adenylate cyclase and guanylate cyclase systems, which can activate other transport proteins, including CFTR.^189^ Furthermore, H_2_S activates and/or upregulates numerous channels and transporters for Ca^2+^,^240^ including Na^+^/Ca^2+^‐exchanger NCX1^241^, ^242^ and the Ca^2+^ permeable TRP channels TRPV6, TRPV4, and TRPV3, all of which are expressed by the apical membrane of the colon.^79^, ^121^, ^190^, ^243^, ^244^ Notably, TRPV3 and TRPV4 are poorly selective and also show permeability to Mg^2+^. Both TRPV3 and TRPV4 are additionally activated by NO via cysteine‐S nitrolysation.^239^ In addition, H_2_S activates TRPM4^190^ while NO activates TRPC1, TRPC4, TRPC5, and TRPV1^239^; other channels may follow.

Interestingly, in a study of rat distal colonic epithelium in Ussing chambers after permeabilization of the apical membrane with nystatin, the H₂S‐donor sodium hydrosulphide was found to enhance basolateral efflux of Ca^2+^ via Na^+^/Ca^2+^‐exchange (NCX1).^242^ In addition, a stimulation of basolateral glibenclamide‐sensitive K^+^ channels was observed. In the intact tissue, this should lead to a lower membrane potential and thus enhance the driving force for the uptake of cations across the apical membrane. While an overall effect on the uptake of Ca^2+^ is debatable given the low intracellular concentration of this ion, effects on the influx of Mg^2+^ should be considerable. On the other hand, the simultaneous stimulation of Cl^−^ secretion might lead to inverse effects.^245^, ^246^

Intriguingly, in vitro data suggest that moderate amounts of H_2_S or NO may have a protective effect on tissues exposed to hypoxia,^247^ while high concentrations may show cytotoxic antiproliferative effects with therapeutic potential in cancer.^248^, ^249^ On the other hand, it must be emphasized that both H_2_S and NO have been implicated both in tumor induction and in the progression of ulcerative colitis and Crohn's disease.^237^ Since microbials have a preference for fermenting fiber, adding prebiotics to the diet is being tried as a promising strategy to limit the production of H_2_S and NO in these patients.^71^

In conjunction, dietary strategies that elevate H_2_S and NO in the gut lumen (e.g. via prebiotic peptides) may have risks that outweigh any potential benefit concerning Ca^2+^ and Mg^2+^ absorption from the gut or bone formation.

### 7.4 Effects of prebiotics on calbindin‐D_9k_, PMCA1b, and NCX1

Calbindin‐D_9k_ is an important marker of intestinal transcellular Ca^2+^ transport in mammals.^39^, ^250^, ^251^, ^252^, ^253^, ^254^ In rats fed a prebiotic, a rise in the transcription of calbindin‐D_9k_ was observed, suggesting enhanced transcellular uptake, although the expression of the Ca^2+^‐ATPase PMCA1b remained unchanged.^255^, ^256^, ^257^ In another study of rats fed an inulin‐oligofructose mixture, increased transcript levels for calbindin‐D_9k_ and for the Na^+^/Ca^2+^‐exchanger NCX1 were observed. Since butyrate in particular serves as a source of energy for the production of ATP by enteric cells, prebiotics should also stimulate the basolateral efflux of Ca^2+^ via pre‐existing PMCA1b pumps.

Notably, calbindin‐D_9k_ does not appear to be the rate‐limiting transport step since in a study of VDR KO mice, higher levels of calbindin‐D_9k_ did not lead to higher levels of absorption.^104^ Furthermore, studies of mice with knockout of the gene for calbindin‐D_9k_ did not have an impaired homeostasis to Ca^2+^ and responded well to 1,25(OH)_2_D3.^115^, ^258^ There is reason to believe that the rate of apical uptake via ion channels will in most cases determine the rate of transport.

### 7.5 Effects of prebiotics and SCFA on the expression of TRPV6

Upregulation of gene expression may contribute to the long‐term stimulation of Ca^2+^ absorption via SCFA. In a colonic cancer cell line (Caco‐2), the addition of SCFA led to an upregulation of mRNA encoding for TRPV6, with significant effects of Na‐butyrate seen at 0.2 mmol L^−1^ and effects maximal at 2 mmol L^−1^.^257^ Effects of Na‐butyrate on Caco‐2 cells reached a maximum at 12 h, after which a decline could be observed. Effects of Na‐propionate were smaller, while Na‐acetate or Na‐lactate did not lead to significant changes in gene expression. Another study of Caco‐2 cells saw no increase in the uptake of Ca^2+^ in response to Na‐butyrate (8 mmol L^−1^) while uptake of Mg^2+^ was reduced.^259^

In mice fed Na‐butyrate, an increase in colonic mRNA for TRPV6 was observed.^25^ The authors did not check if Na‐butyrate reached the colon but noted that colonic pH dropped slightly after feeding Na‐butyrate from 7.3 to ~7, with both values unusually high. Note that feeding Na‐butyrate is not equivalent to feeding prebiotics and cannot be expected to reduce pH, since no protons are produced.

Feeding fructo‐oligo‐saccharides to rats led to increased expression of TRPV6 (and calbindin‐D_9K_) at constant PMCA1b by cells from the colo‐rectal section.^257^ When ovariectomized rats were fed diets containing calcium carbonate or dairy calcium, the addition of inulin (but not lactose) led to a significant increase in the cecal expression of TRPV6 in the dairy group. However, colonic expression of TRPV6 remained unchanged.^27^ In male mice fed xylo‐oligosaccharides, bone mineral density and bone‐breaking strength increased, associated with a decreased caecum pH and increased villus height and length.^260^ Duodenal expression of transcripts for TRPV6 increased. Unfortunately, caecal or colonic epithelia were not investigated.

No significant effects on caecal or colonic TRPV6 expression versus control were observed when mice were fed inulin fibers, despite elevated fecal n‐butyrate with a significant reduction in fecal Ca^2+^ excretion.^22^ Renal Ca^2+^ excretion and renal expression of TRPV5 remained unchanged. In a later part of the same study, the effects of dietary inulin were investigated after inducing hypocalcemia with a proton‐pump inhibitor, leading to a rise in the colonic expression of TRPV6 with a similar trend in the caecum, possibly in response to a hypocalcemia‐induced increase in 1,25(OH)_2_D3. Subsequent feeding of inulin in addition to omeprazole restored serum Ca^2+^, but led to a drop in the colonic expression of TRPV6, again with a similar (but non‐significant) trend in the caecum. In rats fed a prebiotic, expression of TRPV6 remained unchanged in the caecum, dropping slightly in the proximal and distal colon.^31^

### 7.6 Effects of prebiotics and SCFA on the expression of TRPM6 and TRPM7

Very few studies have directly addressed the effects of prebiotics or SCFA on the expression of Mg^2+^ transporters, and the results appear quite variable. An increase in the ubiquitous Mg^2+^ channel TRPM7 was observed in response to feeding rats with Na^+^‐butyrate.^25^ Expression levels of mRNA encoding for the Mg^2+^ channel TRPM6 and for the Na^+^/Mg^2+^‐exchanger CNNM4 remained unchanged. Colonic pH was unusually high (~7), and it is unclear if Na‐butyrate reached the hindgut. As mentioned above, feeding Na‐butyrate is not equivalent to inducing fermentation in the hindgut.

Feeding mice with inulin increased Mg^2+^ absorption and bone stores, in conjunction with an upregulation of TRPM6 and TRPM7 in the hindgut and a reduction of the renal expression of both channels.^60^ A decrease in the renal expression of TRPM6 was confirmed in another study of mice fed inulin. In that study, TRPM6 expression decreased in the caecum with no effect observed in the colon.^22^ No recovery of serum Mg^2+^ occurred in the inulin‐fed mice, which is in contrast to what the same authors saw in humans, where inulin significantly increased serum Mg^2+^ levels and alleviated clinical symptoms in patients suffering from hypomagnesemia.^52^

In another study of rats, a negative correlation of colonic butyrate concentration with plasma Mg^2+^ levels was observed.^259^ The low values of SCFA (acetate <25 mmol L^−1^) suggest that possibly, hindgut fermentation was suppressed, since in healthy animals with adequate fiber, acetate should be >60 mmol L^−1^.^72^, ^73^, ^261^, ^262^ In conjunction, the effect of prebiotics on the caecal and colonic expression of TRPM6 and TRPM7 remains to be clarified.

### 7.7 TRPV3: an alternate pathway for the uptake of Ca^2+^ and Mg^2+^ by the fermentative gut?

TRPV3 is a non‐selective relative of the Ca^2+^‐selective TRPV5 and TRPV6 channels with high sequence similarity across diverse species.^263^, ^264^ TRPV3 was first identified in skin and was originally thought to play a role in thermosensation.^265^, ^266^ However, mutations do not appear to lead to changes in temperature sensing.^267^ In humans, gain‐of‐function mutations lead to severe hyperkeratosis,^268^ while in mice, a hairless phenotype is observed.^269^

TRPV3 is also strongly expressed by the apical membrane of the colon and caecum of humans, pigs, and mice,^79^, ^270^ and by the rumen of cattle and sheep.^271^, ^272^ Based on this expression pattern, a role in the intestinal absorption of cations was suggested over a decade ago.^244^ In a study of pigs, it emerged that only caecum and colon express the full‐length channel protein, although other gastrointestinal tissues express shorter splice variants lacking the pore region.^79^

TRPV3 discriminates poorly between different cations, conducting a plethora of monovalent and divalent cations, including Ca^2+^, Sr^2+^, or even larger cations.^16^, ^160^, ^273^, ^274^ For the bovine and human homologue of TRPV3, this includes the NH_4_^+^ ion (~240pS), the Na^+^ ion (~130pS), the Ca^2+^ ion (~34pS), the NMDG^+^ ion (~34pS),^272^, ^275^, ^276^ and the Mg^2+^ ion (~20pS, own unpublished observations). A conductance to Mg^2+^ is also supported by the fact that Mg^2+^ permeates the structurally similar channels TRPV1, TRPV2, and TRPV4.^160^ Some studies of TRPV3 propose that “pore dilation” can occur,^277^ possibly due to insertion of an additional TRPV3 subunit into the classical tetrameric assembly.^274^

The single‐channel conductance for monovalent cations is reduced by divalent cations such as Ca^2+^ and Mg^2+^ from both sides of the membrane,^275^ related to an acidic D641 residue in the extracellular pore loop and two acidic residues (E679, E682) in the inner pore region.^277^, ^278^, ^279^ The existence of two separate binding sites is supported by our own study of HEK‐293 cells overexpressing the bovine homologue, which suggests that while extracellular divalents lead to rectification of monovalent currents, intracellular application does not.^275^ In that study,^275^ divalent cations were also able to block the initially small inward currents observed in NMDG^+^ containing solutions. The NMDG‐induced whole cell currents rose considerably with time. However, in single‐channel experiments, we did not observe an increase in conductance suggesting pore dilation, although the open probability rose with the duration of the experiment.

Intriguingly, herbal compounds such as menthol and thymol activate TRPV3.^280^, ^281^ Both agents stimulate the uptake of cations by the ruminal epithelium in Ussing chambers^232^, ^271^, ^272^, ^282^, ^283^ and improved Ca^2+^ homeostasis was observed in ruminants in vivo that were fed a feed additive containing menthol among other ingredients.^234^, ^283^, ^284^ Note that the menthol‐sensitive channel TRPM8 is not expressed by the rumen, while expression of TRPA1 is very weak or lacking.^232^, ^234^, ^282^

**FIGURE 6:**
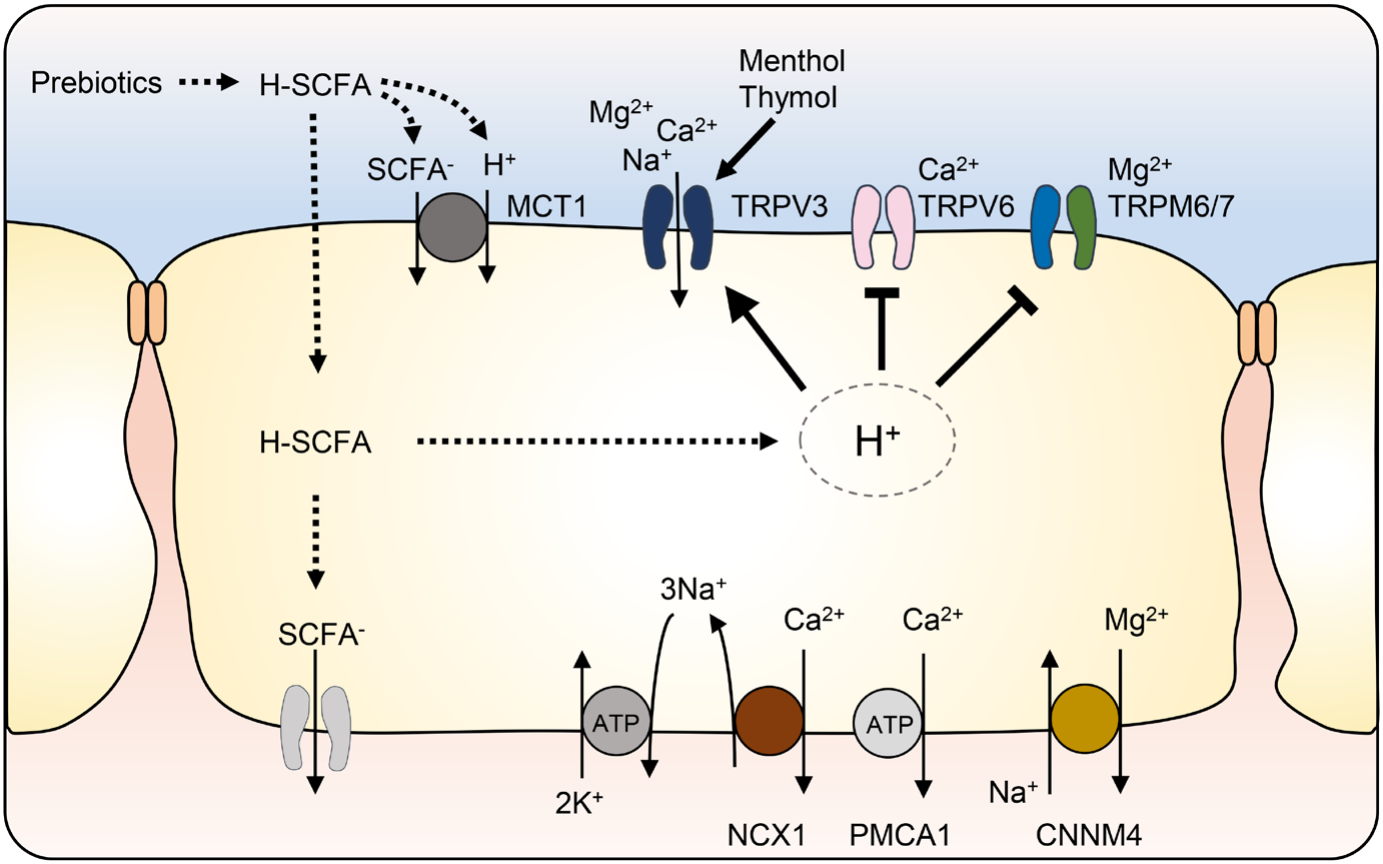
Model for the effect of prebiotics on channels mediating the absorption of Ca^2+^ and Mg^2+^. Prebiotics lead to an increased production of SCFA. SCFA are taken up into the epithelium via various mechanisms (e. g., diffusion, anion exchange, co‐transport with protons via MCT1) with cytosolic release of protons. While TRPV6 and TRPM6/TRPM7 are blocked by cytosolic protons, the non‐selective cation channel TRPV3 is activated by intracellular acidification, allowing an increased uptake of various cations, including Na^+^, NH_4_^+^, Ca^2+^, and Mg^2+^.

In contrast to TRPV6 and TRPM6/TRPM7, TRPV3 is activated by intracellular protons involving an N‐terminal histidine, His‐426, with activation maximal at pH 5.5.^285^, ^286^ Accordingly, exposure to weak acids such as glycolic acid^285^ or butyric acid^271^ activates both the influx of monovalent cations and of Ca^2+^ into cells expressing TRPV3 (Figure 6). Furthermore, TRPV3 contains cysteine (Cys) residues and can be activated by H_2_S or NO.^190^, ^238^, ^239^ TRPV3 is thus a promising candidate gene that might explain why enhancing fermentational activity can lead to increases in the active transport of Ca^2+^ and Mg^2+^ by the fermentative parts of the gut. It is intriguing to speculate that this pathway might be activated by compounds freed from herbs such as thyme leaves or cinnamon bark after fermentation in the colon. Indeed, spices may be more important for human nutrition than is frequently believed.^287^

A further poorly selective cation channel, TRPV4, is also strongly expressed by the rumen and the colon.^79^, ^121^, ^232^, ^234^, ^282^ Like TRPV3, TRPV4 is activated by H_2_S or NO.^239^, ^243^ Unlike TRPV3, the channel is ubiquitously expressed, and a role in osmoregulation has been suggested.^288^, ^289^ While menthol or thymol do not activate TRPV4, an influx of SCFA might lead to cell swelling with activation of the channel. A certain role in SCFA‐induced Ca^2+^ and Mg^2+^ uptake thus appears possible. It should be stressed that there is evidence for the involvement of further nonselective members of the TRP channel family in the uptake of cations by the hindgut,^290^ and that the role that these channels play in intestinal physiology is only gradually emerging.

## 8 CONCLUSIONS

Stimulation of fermentational activity by dietary prebiotics stimulates the uptake of Ca^2+^ and Mg^2+^ across the hindgut via mechanisms that remain to be clarified. Despite the progress made in the past decades, it is becoming clear that the story of how these two minerals are absorbed is more complex than previously thought. A better understanding should open new possibilities for prevention and therapy of disturbances in Ca^2+^ and Mg^2+^ homeostasis–a topic that is likely to become increasingly more important as populations age while increasing numbers of young adults turn away from milk and milk products as the traditional main source of these important minerals.

## AUTHOR CONTRIBUTIONS

**Friederike Stumpff:** Conceptualization; supervision; writing – original draft; funding acquisition. **David Manneck:** Writing – review and editing; validation; visualization.

## CONFLICT OF INTEREST STATEMENT

The authors declare no conflicts of interest.

## Data Availability

No new data were generated within the context of this review article based on the references cited below.
